# Early Ordovician sea scorpions from Morocco suggest Cambrian origins and main diversification of Eurypterida

**DOI:** 10.1098/rspb.2025.2061

**Published:** 2025-11-12

**Authors:** Peter Van Roy, Jared C. Richards, Javier Ortega-Hernández

**Affiliations:** ^1^ Department of Geology (WE13), Ghent University, 9000 Ghent, Belgium; ^2^ Czech Geological Survey, Klárov 3, 118 21 Prague 1, Czech Republic; ^3^ Museum of Comparative Zoology and Department of Organismic and Evolutionary Biology, Harvard University, Cambridge, MA 02138, USA

**Keywords:** Arachnida, Cambrian explosion, Carcinosomatidae, Chelicerata, Eurypterida, Fezouata Biota, Great Ordovician Biodiversification Event, Konservat-Lagerstätte, Pterygotidae, Tremadocian

## Abstract

Eurypterida were a diverse clade of aquatic euchelicerates that occupied environments ranging from freshwater to fully marine and included several of the largest euarthropods on record. Although a Middle Ordovician megalograptid hitherto represented the oldest evidence of this clade, its phylogenetic position suggested an earlier history for the origin and main diversification within Eurypterida. Here, we report unequivocal eurypterid fragments from the Early Ordovician Fezouata Biota of Morocco, pre-dating the previously oldest record of this group by 12–15 million years. We describe ?*Carcinosoma aurorae* n. sp. based on several distinctively spinose isolated appendages diagnostic of the eurypterine clade Carcinosomatidae. This discovery demonstrates that the major morphological and ecological diversifications within Eurypterida between swimming Eurypterina and benthic crawling Stylonurina had taken place by the Early Ordovician. Furthermore, the derived phylogenetic position of carcinosomatids implies that most eurypterine clades had already diversified by that time. A cuticle patch with dense scales, reminiscent of pterygotids, likely belongs to a second eurypterid species. The remarkable diversity of euchelicerates in the Fezouata Biota indicates undocumented Cambrian origins and provides further evidence for an early eurypterid radiation centred off Gondwana. Significantly, the sister-group relationship between Eurypterida and Arachnida entails equally early arachnid origins.

## 1. Introduction

Eurypterida, or ‘sea scorpions’, is a major extinct clade of aquatic euchelicerates and represents one of the most iconic post-Cambrian Palaeozoic invertebrate groups, including several of the largest euarthropods to have ever existed [1–5]. Eurypterids had a wide-ranging distribution and reached their peak diversity during the Silurian [6]. Over their evolutionary history, they played significant ecological roles, including as top predators, scavengers and giant sweep-feeders in diverse aquatic environments ranging from freshwater habitats over brackish lagoonal settings to fully open marine environments [5–9], with some species being capable of limited subaerial excursions [5,8,10–13]. Eurypterida is divided into two major clades: the Eurypterina, characterized by their last prosomal appendage pair modified into swimming paddles, and the Stylonurina, which exclusively had walking limbs for benthic locomotion [5,7,14,15]. The oldest previously described eurypterid, and also the oldest eurypterine, is the megalograptid *Pentecopterus decorahensis* [14], from the Darriwilian (Mid Ordovician) Winneshiek Shale of Iowa, USA. Stylonurines appeared later, with the rhenopterid *Brachyopterus stubblefieldi* [16] from the Sandbian (Late Ordovician) of Wales, UK. Eurypterid diversity and ecological morphospace occupation started to decline from the Devonian onwards [6] until their ultimate demise during the Permian. The last eurypterine, the adelophthalmid *Adelophthalmus sellardsi* [17] from Kansas, USA, disappeared in the Artinskian (Permian, Cisuralian). Stylonurines, represented by the mycteropoid ?*Woodwardopterus freemanorum* [18] from Queensland, Australia, ultimately followed during the Changhsingian (terminal Permian, Lopingian). However, see [5] for the suggestion that this fossil may potentially belong to a scorpion instead, in which case *Campylocephalus permianus* [19] and *Campylocephalus oculatus* [20] from the late Kungurian–Roadian of Russia represent the youngest eurypterids.

**Figure 1.**
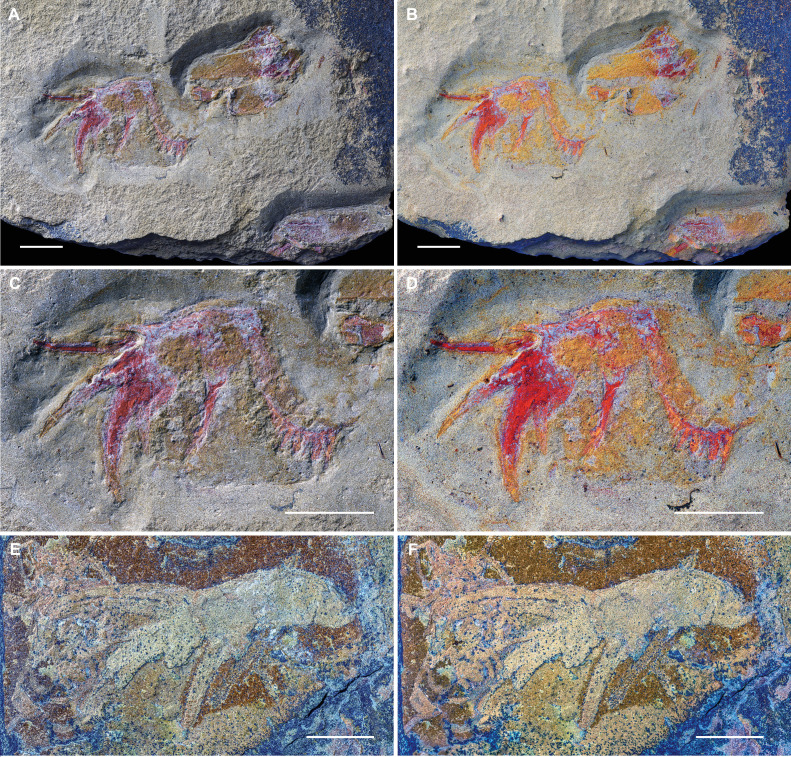
Isolated appendages of ?*Carcinosoma aurorae* n. sp. from the Early Ordovician (upper Tremadocian) Fezouata Biota of Morocco. (A–D) Holotype NM S 5974 consisting of a walking limb, gnathobase and four fragmentary tergites, part. (A) Overview, dry. (B) Overview, under a mixture of ethanol and propanol. (C) Details of the limb and gnathobase, dry. (D) Details of the limb and gnathobase, under a mixture of ethanol and propanol. (E,F) Paratype MCZ.IP. 202866, consisting of nearly complete walking limb, part. (E) Dry. (F) Under demineralized water. All scale bars represent 10 mm.

**Figure 2.**
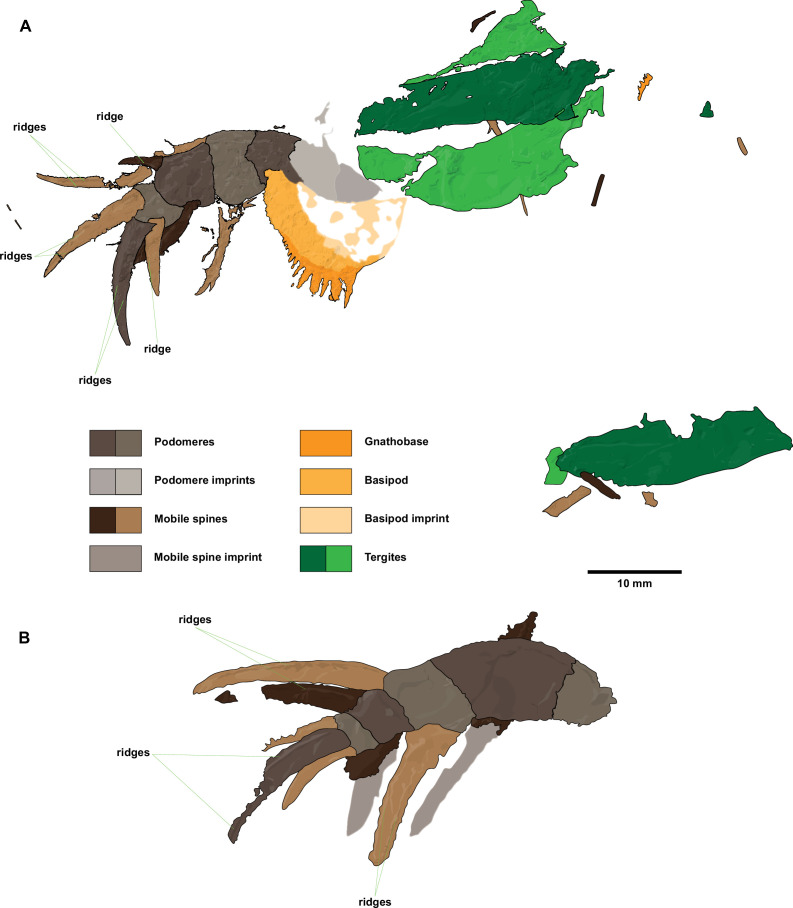
Interpretative drawings of ?*Carcinosoma aurorae* n. sp. appendages. (A) Holotype NM S 5974. (B) Paratype MCZ.IP. 202866. Scale bar represents 10 mm.

**Figure 3.**
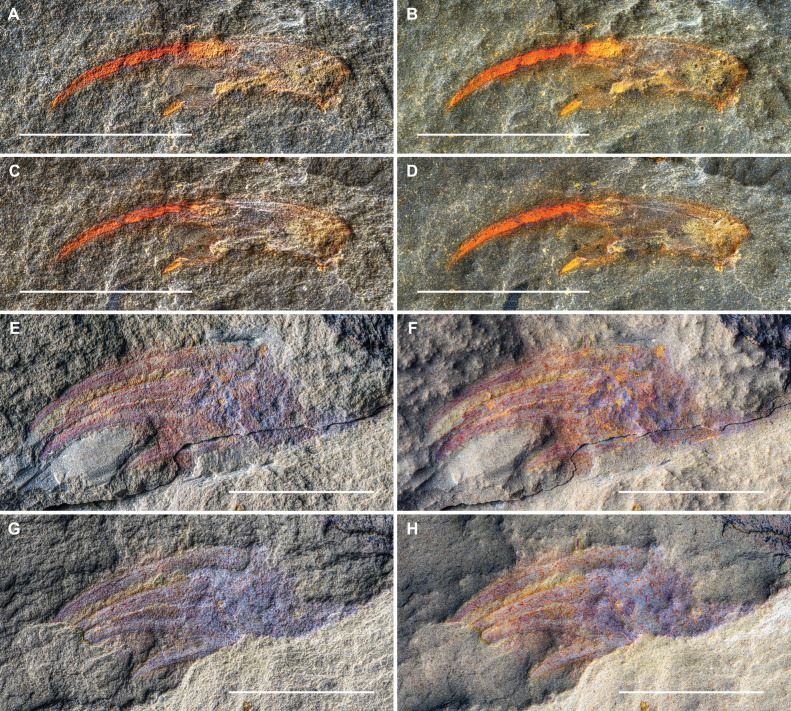
Appendage terminations of ?*Carcinosoma aurorae* n. sp. from the Early Ordovician (upper Tremadocian) Fezouata Biota of Morocco. (A–D) Specimen SC 148, consisting of the distal termination of appendage. (A) Part, dry. (B) Part under water. (C,D) Counterpart, lighting from SW and mirrored to create a false-positive relief image. (C) Dry. (D) Under water. (E–H) Specimen NM S 5985, consisting of the distal termination of appendage. (E) Part, dry. (F) Part, under water. (G,H) Counterpart, lighting from SW and mirrored to create a false-positive relief image. (G) Dry. (H) Under water. All scale bars represent 10 mm.

**Figure 4.**
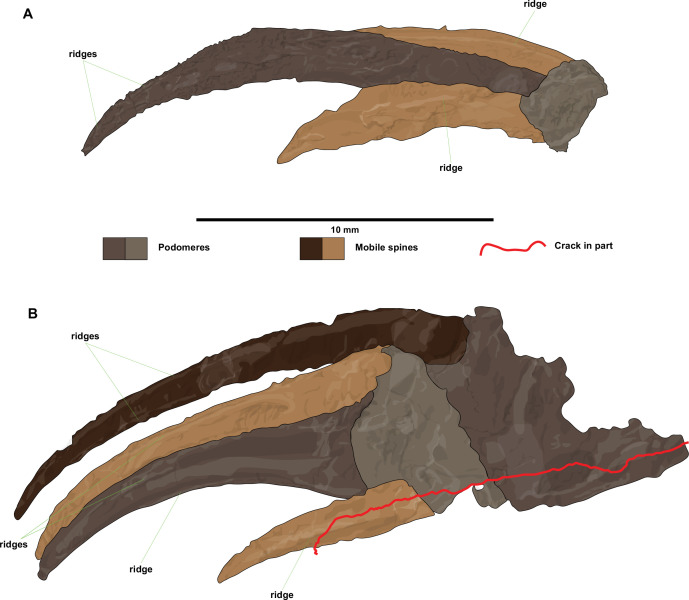
Interpretative drawings of ?*Carcinosoma aurorae* n. sp. appendage terminations. (A) Interpretative drawing of SC 148. (B) Interpretative drawing of NM S 5985. Drawings combine information from both the part and the counterpart. Scale bar represents 10 mm.

**Figure 5.**
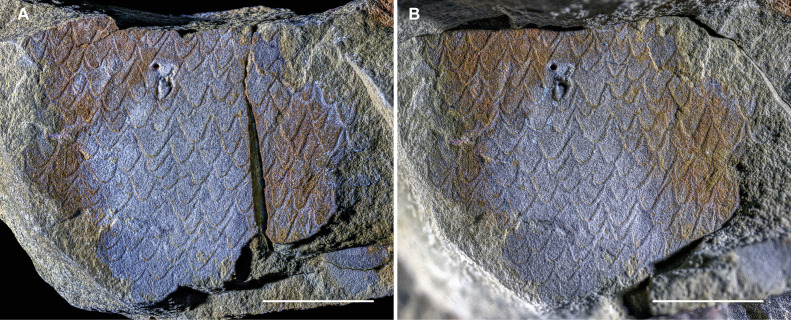
Isolated ornamented eurypterid cuticle from the Early Ordovician (upper Tremadocian) Fezouata Biota of Morocco. (A,B) MCZ.IP. 202867, patch of cuticle, showing dense linguoid ornament. (A) Part, dry. (B) Counterpart, dry, lighting from SW and mirrored to create a false-positive relief image. Scale bars represent 10 mm.

Classic, morphology-based cladistic analyses indicate that eurypterids are the sister group of Arachnida, which includes all extant clades of terrestrial chelicerates [14,15,21–23]. Conversely, a recent extensive whole-evidence study involving both morphological and molecular characters [24,25] resurrected Merostomata, essentially a grouping of eurypterids, chasmataspidids and xiphosurids, placed together with ricinuleids as a sister group to the Arachnopulmonata clade of arachnids, in effect dismantling the classical concept of Arachnida. Regardless of which scheme is correct, it is clear that the phylogenetic position of eurypterids makes them significant for understanding the timing of major events in the early diversification of crown-group euchelicerates during the Early Palaeozoic, and therefore constraining the timing for their origins directly impacts our view of the post-Cambrian biosphere. Although the Mid to Late Ordovician fossil record suggests that eurypterids had evolved at least by the Early Ordovician, and possibly as early as the late Cambrian [26,27], the absence of direct fossil evidence has hampered a more precise timing for their origins and early diversification until now. Here, we report the discovery of unequivocal eurypterid remains from the late Tremadocian (Early Ordovician) Fezouata Biota of Morocco (electronic supplementary material, figure S1). The specimens correspond to spinose appendages (figures 1–4), characteristic of the eurypterine clade Carcinosomatidae (electronic supplementary material, figures S2, S3 and S4A–D), highly derived forms [5,14,15,21] characterized by their flat, scorpion-like bodies and a curved styliform telson (see electronic supplementary material), and a large patch of cuticle showing a highly typical eurypterid ornament of strongly pronounced linguoid scales (figure 5), evocative of—but by itself not conclusive for—pterygotids (electronic supplementary material, figure S4E).

## 2. Systematic palaeontology

Owing to the incomplete preservation of the appendages and some uncertainty about the podomere count of carcinosomatid walking limbs, podomeres are simply numbered starting from the most proximal preserved podomere. Because the original orientation of disarticulated appendages cannot be established, ‘upper’, ‘lower’, ‘top’ and ‘bottom’ refer to the position of structures with reference to their position and orientation in the photographs of the specimens.

Euarthropoda Lankester, 1904 [28] (*sensu* [29])
  Euchelicerata Weygoldt & Paulus, 1979 [30]
  Prosomapoda Lamsdell, 2013 [31]
  Planaterga Lamsdell, 2013 [31]
  Dekatriata Lamsdell, 2013 [31]
  Eurypterida Huxley, 1857 [32]
  Eurypterina Novojilov, 1962 [33]
  Diploperculata Lamsdell Hoşgör & Selden, 2013 [34]
  Carcinosomatoidea Størmer, 1934 [35]
  Carcinosomatidae Størmer, 1934 [35]
  Genus: ?*Carcinosoma* Claypole, 1890 [36]
  ?*Carcinosoma aurorae* n. sp.

*LSID*. urn:lsid:zoobank.org:pub:4233D8FE-C6EE-485E-981D-9001013DD58A

*Derivation of name*. Genitive, from Latin, *aurora*, *-ae*, meaning ‘of the dawn’, referring to the early occurrence of this animal, making it the oldest eurypterid currently on record.

*Holotype*. NM S 5974 (part only).

*Paratype*. MCZ.IP. 202866 (part only).

*Additional specimens*. SC 148 (part A and counterpart B), NM S 5985 (part a and counterpart b).

*Diagnosis*. Species of ?*Carcinosoma* with articulating spine pairs of unequal length on podomeres of limbs II–V , with positions of long and short spines alternating between podomeres. Gnathobases with long and slender teeth.

*Description*. NM S 5974 is composed of the distal five podomeres of a walking limb, a gnathobase and four partial tergites together with some smaller cuticular fragments (figures 1A–D and 2A). Only the part is available. The walking limb is preserved in dorso-lateral aspect and has a total preserved sagittal length measured along the curve of *ca* 33 mm. Podomeres 2–5 are completely preserved, while the remains of the most proximal preserved podomere are more fragmentary; a vague imprint of one or two even more proximal podomeres may be present. Podomeres 2–4 differ in terms of their measured proportions, with 3 being the longest and 4 the shortest (electronic supplementary material, table S1). The terminal podomere 5 is developed into a robust, downward-curving spine. Podomeres 2–4 all carry a pair of strong, highly developed, mobile ventro-lateral spines distally. The mobile spines on each podomere differ in size. The position of the long and short spines alternates from podomere to podomere along the length of the appendage. No spines are evident on the poorly preserved proximal podomere. There is also some variability in terms of spine morphology, with podomere 2 having the longest spines, podomere 3 the shortest and podomere 4 the most robust mobile spines; the latter have some evidence for the presence of longitudinal ridges. The gnathobase, likely belonging to the basipod associated with the appendage, is well preserved and flipped horizontally relative to the limb. The gnathobase has a rounded, serrated edge with long, narrow teeth that become progressively larger distally, the smallest discernible tooth being *ca* 0.4 mm long and *ca* 0.3 mm wide at its base, with the longest tooth measuring *ca* 3.3 mm long and 0.9 mm wide at its base. The penultimate tooth is the widest, at *ca* 1.3 mm wide at its base and 2.8 mm in length. A total of some dozen teeth may be discerned. The less sclerotized parts of the basipod are not clearly preserved, but staining of the rock suggests a rounded to sub-rectangular outline. Three incomplete and wrinkled tergites are round to the right of the appendage, with the top one being mostly hidden below the other two. The upper tergites appear to be short and almost straight. The lower tergite appears to be largely complete, missing only its distal margins; it has a similar length to the preceding tergites, but it displays a moderate curvature. Near the edge of the slab is a fourth partial tergite.

MCZ.IP. 202866 is a relatively poorly preserved but largely complete appendage composed of six podomeres; overgrowth of a thin decay halo complicates detailed measurement and identification of fine surface features (figures 1E,F and 2B). Only the part is available. The appendage is exposed in dorso-lateral view and has a total preserved length along the curve of *ca* 49.5 mm. The basal first podomere widens distally and is devoid of spines. Podomere 2 is the longest and widest podomere, after which the appendage gradually tapers distally. The penultimate podomere 5 is the shortest, being only about half as long as wide. The terminal sixth podomere is developed into a long, robust, curved spine with longitudinal ridges on its surface; the distal part of the podomere is broken away. Podomeres 2–5 all carry a pair of robust spines, which are longest on podomere 3. On podomere 2, only the bases of the spines and the imprint of the lower spine are preserved. The spines of podomere 3 are largely complete. On podomere 4, the upper spine is largely complete, while the base of the lower spine and a partial imprint are preserved. The lower spine of podomere 5 is complete, while the upper spine misses its distal portion. Several of the spines show evidence for the presence of longitudinal ridges. Measurements of the podomeres and spines are provided in electronic supplementary material, table S2.

SC 148 corresponds to the terminal part of a walking limb, consisting of the last two podomeres, in lateral view, with both part and counterpart available (figures 3A–D and 4A). The terminal podomere is a long and very robust spine. The penultimate podomere is short and shows one heavy mobile spine pair inserting distally in a ventro-lateral position, flanking the terminal podomere. Both the terminal podomere and the mobile spines show evidence of longitudinal ridges on their surface. Measurements of podomeres and spines are provided in electronic supplementary material, table S3.

NM S 5985 preserves the terminal three podomeres of a walking limb in ventral view, with both part and counterpart available (figures 3E–H and 4B). The most proximal podomere is incompletely preserved. The terminal podomere takes the shape of a massive spine that expands considerably at its base. The penultimate podomere is short and carries a pair of mobile ventro-lateral spines distally, with one spine being almost twice as long as the other. The proximal-most podomere that is preserved is incomplete, but it does preserve one long, mobile ventro-lateral spine distally. Measurements of podomeres and spines are provided in electronic supplementary material, table S4.

*Occurrence*. Early Ordovician, late Tremadocian, *Sagenograptus murrayi* graptolite biozone, Fezouata Biota, east of Bni Zoli, southeastern Morocco (electronic supplementary material, figure S1).

Eurypterida Huxley, 1857 [32]

*Description*. MCZ.IP. 202867 represents a roughly sub-rectangular-shaped patch of cuticle, about 30 mm wide and 25 mm high (figure 5). Both part and counterpart are available, the part being broken vertically and re-glued on its right side. The surface of the cuticle shows a dense ornament of large, partially overlapping linguoid scales of approximately equal size. Individual scales are about as long as they are wide at their base, varying roughly between 2 and 3 mm in both directions.

*Occurrence*. Early Ordovician, late Tremadocian, *Sagenograptus murrayi* graptolite biozone, Fezouata Biota, east of Bni Zoli, southeastern Morocco (electronic supplementary material, figure S1).

## 3. Discussion and conclusions

### (a) Interpretation and reconstruction of ?*Carcinosoma aurorae*

The new Fezouata appendages showcase a robust morphology, with most observed podomeres (except for the terminal podomere and the second podomere of MCZ.IP. 202866) having proportions that are at least as high as they are long. Taken together, the specimens preserved in ventral (NM S 5985; figure 3E–H), dorso-lateral (NM S 5974 and MCZ.IP. 202866; figure 1) and lateral (SC 148; figure 3A–D) views show that the appendage podomeres had a circular cross-section. The terminal podomere also had a circular cross-section and took the shape of a long, downward-curved and heavy spine with a longitudinally striated surface. MCZ.IP. 202866 shows that, except for the basal and terminal podomeres, all intervening podomeres carried long, robust mobile spines set into distally located ventro-lateral sockets; the absence of spines on the most basal preserved podomere in NM S 5974 likely results from the disarticulation or non-preservation of the mobile spines; alternatively, it is possible this podomere truly lacked spines on some limbs in the series. The mobile spines too have a longitudinally striated texture. The spines on each podomere are of unequal length, with the positions of shorter and longer spines alternating between podomeres. The spines show a moderate downward curvature, except for the very long and poorly preserved upper spine of podomere 2 in NM S 5974 (figures 1A–D and 2A); the latter upward curvature is likely a taphonomic artefact resulting from increased pliability of sclerotized structures owing to the decay of the biopolymers composing the cuticle [37,38]. It is also important to note that NM S 5974 likely represents a moult (see discussion below), which would further increase the flexibility of normally rigid structures. Owing to the poor preservation of the proximal part of the appendage in NM S 5974, and the incomplete nature of NM S 5985 and SC 148, it is impossible to give a total podomere count for these appendages. However, the vague imprint of the proximal part that is preserved in NM S 5974 suggests one or two more podomeres, giving a total count of six or seven podomeres. MCZ.IP. 202866 appears to be essentially complete and provides a count of six limb podomeres (figures 1E,F and 2B). The poor preservation of the proximal portions of the limb and of the basipod other than the heavily sclerotized edge of the gnathobase also makes it impossible to say with certainty whether these two elements were still attached to each other in NM S 5974; this, however, seems plausible, in which case the limb is preserved folded back over the basipod. The curved gnathobase itself is characterized by its heavy dentition, with the pointed and slender teeth becoming increasingly long distally. The association of both straight and curved slender tergites in NM S 5974 suggests the tergites of the trunk became increasingly recurved towards the posterior. All the appendages and their associated fragments point to a relatively small animal, probably no larger than 20–30 cm; however, it is impossible to ascertain whether the specimens represent juveniles or adults based on the available material. In this respect, it is worth noting that the relatively poor state of preservation, crumpled and jumbled appearance of the tergites, and possible folding of the limb over the basipod in NM S 5974 suggest that this specimen likely represents a moult. The heavy armature of spines on the limbs and the strongly serrated gnathobase leave little doubt that they were raptorial, and, like other carcinosomatids, ?*Carcinosoma aurorae* was a predator.

### (b) Phylogenetic affinities

#### (i) Isolated appendages

The highly spinose nature of the limbs of ?*Carcinosoma aurorae* superficially resembles the arthropodized frontal appendages of radiodonts, ecologically diverse stem-group euarthropods commonly documented from Cambrian deposits with exceptional preservation [39,40] but now known to persist well into the Ordovician [41–47]. Although several radiodonts have subchelate appendages, particularly well expressed among amplectobeluids [48–51], they lack a terminal podomere that is developed into an extremely elongated spine as observed in the Fezouata appendages (figures 1–4). The position of the ventral endites in radiodonts is typically medial within the podomere, when contrasted with the far anterior insertion point observed in the new Fezouata appendages. Furthermore, the association of the Fezouata appendages with a typical curved euarthropod gnathobase, morphologically distinct from the gnathobase-like structures in either amplectobeluids or other radiodonts [48,50,52], and the presence of sclerotized tergites in NM S 5974 (figures 1A,B and 2A) all argue against a radiodont interpretation of the material.

The appendages of ?*Carcinosoma aurorae* fully conform to those of eurypterids, which plesiomorphically have pairs of ventral spines on the limb podomeres. A first comparison can be drawn to megalograptids, and to *Pentecopterus decorahensis* in particular based on the resemblance of the first three post-cheliceral appendages (appendages II–IV) showing similar extreme spinosity [14]. However, the distal spine-bearing podomeres in *Pentecopterus* carry two pairs of spines: an antero-laterally positioned fixed pair of enlarged spines and a postero-ventral pair of smaller, robust, mobile spines. This is considerably different from the organization found in ?*Carcinosoma aurorae*, where no fixed spines are present, and only a single pair of greatly enlarged mobile spines is located on the podomeres antero-ventrally. In general, megalograptids typically bear more than one pair of spines on the distal podomeres of their hyper-spinose appendages II–IV [5,14,53–55], and for this reason, a megalograptid affinity for the material can be rejected. On the other hand, the arrangement found in ?*Carcinosoma aurorae* does closely match the extremely spinose morphology of carcinosomatids, which have long, paired mobile spines inserting distally on their podomeres [5,56–59], and have the terminal podomere developed into an extremely long and heavy spine. The slight upward curvature of the long upper spine on podomere 2 of NM S 5974 is unusual but likely represents a taphonomic artefact (see above). The attribution to Carcinosomatidae finds further support in the morphology of the gnathobase of NM S 5974, which matches the long and slender teeth known from other carcinosomatids [4,59,60] (electronic supplementary material, figures S2C and S4C). A further significant indication is the presence of both straight and slightly curved, slender tergites in the disarticulated assemblage NM S 5974: the tergites of the broad, flat preabdomen of carcinosomatids generally tend to become progressively recurved towards the posterior; the curved tergite in the assemblage likely represents the final preabdominal tergite (=tergite 7, somite XIV, first metasomal tergite). While by itself not strictly diagnostic, the longitudinal striations on the spines and terminal podomere are also indicative of a carcinosomatid affinity for the material.

Within Carcinosomatidae, the walking limbs of ?*Carcinosoma aurorae* are most similar to those of *Carcinosoma*, with which they share the robust morphology and particularly heavy spines (electronic supplementary material, figures S2A,B, S3 and S4A,B; see also Fig. 60C,D in [5]). Conversely, the limbs and spines of both *Rhinocarcinosoma* and *Eusarcana* are more delicate [5,54,56,57,61], while the recently established genus *Cruinnopterus* [5] has multiple spine pairs on its proximal podomeres, and only two podomeres, each carrying a pair of spines, have been described for the supposedly basal carcinosomatid *Holmipterus* [62]; the walking limbs of *Tigrisopterus* are unknown [63]. ?*Carcinosoma aurorae* is distinguished from any previously described species by the uneven length of spine pairs on the podomeres and the alternation of this character along the appendage. Because of the incomplete nature of the available specimens, we chose to only tentatively assign them to the genus *Carcinosoma*, pending the discovery of more complete material.

Specimens SC 148 and NM S 5985 are somewhat less straightforward to attribute to Carcinosomatidae because of their incompleteness (figures 3 and 4). The presence of well developed spines might evoke similarities with the great appendages of megacheirans, a group of non-biomineralized euarthropods typified by the presence of well developed raptorial limbs, known from Cambrian to Silurian (possibly Devonian) sites with exceptional preservation. This comparison can be readily falsified. The spines in SC 148 and NM S 5985 are both elongate and distinctively curved (figures 3 and 4); among megacheirans, the leanchoiliids have similarly elongate spines [64–66], but these are consistently straight in all known taxa and frequently associated with a terminal flagellum, both characters absent from the Fezouata material. Furthermore, all megacheirans only have one anteriorly pointing fixed spine per podomere, with all spines arranged on the same (ventral) side of the raptorial appendage in a step-wise manner that corresponds to the relative position of the three or four distal-most podomeres [67–70]. In both SC 148 and NM S 5985, there are clear divisions and broken cuticle separating the spines from their podomeres that indicate their basal articulation site (figures 3 and 4), whereas most megacheiran spines are extensions of the anterior podomere and thus lack an articulation [67–70]. Moreover, both SC 148 and NM S 5985 clearly show that two spines of different lengths attach to the penultimate podomere flanking the terminal spinose podomere, which is inconsistent with the raptorial appendages of all megacheirans described to date. In conclusion, we consider that, despite their fragmentary nature, the morphology of both these appendage terminations fully conforms to the more complete specimens NM S 5974 and MCZ.IP. 202866, and they are hence included among the material of ?*Carcinosoma aurorae*.

#### (ii) Cuticle

The striking ornament of the cuticle of specimen MCZ.IP. 202867 may invite a first comparison to the very similar cuticular ornament found on the carapace and abdominal sclerites of some phyllocaridids [71–73]. A problem with this interpretation is that archaeostracan carapace valves and abdominal sclerites have a considerable degree of convexity, at least some of which would generally be expected to be retained in Fezouata; instead, the actual specimen is essentially flat, while retaining the ornament in pronounced relief, which rather suggests that it represents a fragment of a tergite of a large dorso-ventrally flattened euarthropod. In addition, the significant size of the cuticular fragment, which is not bounded by any natural margin on any side, would require a very sizeable phyllocaridid to accommodate, which seems unlikely; although, admittedly, the Silurian *Ceratiocaris papilio* did achieve large sizes [72,73]. All considered, the strongly developed linguoid scales, flat appearance, and large size of MCZ.IP. 202867 (figure 5) are a considerably better match for Eurypterida—the other euarthropod clade widely documented to develop this highly distinctive ornament. However, this type of ornament is of restricted use for detailed systematics because it is widely shared among all eurypterid clades, and taxa generally display multiple types of ornament that may grade into each other [55]. Therefore, beyond a definitive eurypterid affinity, it is not possible to attribute the patch of cuticle to any specific clade. Carcinosomatids have scales [5,56,57,60,74], and if MCZ.IP. 202867 belongs to ?*Carcinosoma aurorae*, the considerable dimensions of this cuticle fragment would indicate that the new species attained a significantly greater size than the 20–30 cm range suggested by the isolated appendage specimens alone. However, carcinosomatid ornamentation typically consists of relatively fine, small and dispersed scales (electronic supplementary material, figure S4D). Therefore, the large size of the cuticular fragment and its dense, coarse ornament indicate that it likely belongs to a different, much larger eurypterid that shared the Early Ordovician Fezouata seas with the comparatively small ?*Carcinosoma aurorae*. Indeed, the Fezouata cuticle fragment with well developed linguoid scales evokes the densely scaled cuticle of pterygotids (electronic supplementary material, figure S4E); however, it is impossible to firmly make this identification based on ornament alone, as the presence of scales is widespread among all eurypterid clades [5,55].

### (c) Rarity of eurypterids in the Fezouata Biota

Eurypterids typically have significantly sclerotized exoskeletons and should in principle have good fossilization potential within the exceptionally preserved Fezouata Biota given the abundance of diverse non-biomineralized euarthropods in the fauna [41,43,44,46,47,75–81]. However, to date, no complete eurypterid specimens have been described from this deposit. NM S 5974 and MCZ.IP. 202867 are the most compelling evidence for the presence of eurypterids in the Early Ordovician so far. However, over the past 15 years, multiple distal carcinosomatid limb fragments, usually consisting of the last two podomeres, have been collected from the Fezouata Shales. We hypothesize that the powerful swimming capabilities of eurypterines would have allowed them to escape sediment clouds like those that buried the benthic Fezouata fauna, thus making their remains extremely scarce in this deposit. The fragmentary specimens recovered so far likely originated from carcasses that decayed while floating in the water column, with pieces falling off and making their way down to the sea floor, or from moults, with NM S 5974 likely representing the latter. In the context of ?*Carcinosoma aurorae*, it is instructive to note that carcinosomatid fossils are rare, and relatively complete articulated individuals are even more infrequent. This is likely due to the highly free-swimming marine open-water autecology of this group, which often co-occurs with the ocean-going pterygotids [4,8,61], indicating that they were capable of spending significant time in the water column (see also electronic supplementary material).

### (d) Eurypterids and the timing of early euchelicerate diversification

Until now, *Pentecopterus decorahensis* [14] from the Middle Ordovician (*ca* 467–464 Ma) Winneshiek Shale [82] was regarded as the oldest eurypterid in the fossil record. The lower interval of exceptional preservation of the Fezouata Biota sits near the top of the *Sagenograptus murrayi* graptolite biozone [83], dated to 479 Ma [84], which means that the Fezouata Biota hosts the earliest known eurypterids, extending their temporal range by approximately 12–15 Myr relative to *P. decorahensis*. Furthermore, the likelihood that the cuticle fragment MCZ.IP. 202867 and the limb specimens may belong to different eurypterid species would in turn make Fezouata the first Ordovician locality with more than one eurypterid genus.

The Fezouata Biota is home to a remarkable diversity of both stem and crown euchelicerates [41,75,79] (P. Van Roy 2007–2025, personal observations), suggesting that this group may have first radiated off Gondwana, possibly as part of the initial phase of the Great Ordovician Biodiversification Event (GOBE). This further implies that euchelicerates have a significant undocumented early history, likely reaching back well into the Cambrian. The reason for the absence of euchelicerates from the Cambrian macrofossil record may result from the group occupying shallow marine environments, which are generally not captured by the window of Cambrian Burgess Shale-type preservation [85,86], resulting in their early radiation remaining undocumented. This idea is supported by the recent discovery of likely offacolid and chasmataspidid fossils from tropical lagoonal deposits from the late Cambrian of Siberia [26,27]. The notion of a largely undocumented marine radiation in shallow marine Cambrian environments is also suggested by several shallow-water small carbonaceous fossil (SCF) biotas, which hint at the presence of considerably more modern faunas than those preserved in deeper-water Burgess Shale-type settings [87–92]. The recent discovery of a deeper marine SCF biota showing a comparable composition to that of Burgess Shale-type faunas from a similar setting confirms that the differences between shallow-water SCF and deeper-water Burgess Shale-type assemblages are not a taphonomic artefact, but represent a genuine signal [93]. The case for a shallow-water origin and radiation of euchelicerates is also supported by the well established fact that, generally, shallow-water faunas tend to be cradles of evolutionary innovation, whereas deeper- and colder-water habitats tend to be more conservative and act as evolutionary refugia [94–98], with a distinct negative diversity gradient running from the Equator to the Poles [99,100] (but see [101] for a more nuanced view).

?*Carcinosoma aurorae* is a member of the eurypterine clade Carcinosomatidae, which carries the significant implication that the fundamental split between the swimming eurypterines and the crawling stylonurines pre-dates the Fezouata fossils. Carcinosomatids are among the most phylogenetically derived eurypterine clades [5,14,15,21], which further implies that all major eurypterine clades, with the possible exception of pterygotoids and adelophthalmids, had already diversified by the Tremadocian (Early Ordovician). Furthermore, pending confirmation that the cuticle fragment MCZ.IP. 202867 could belong to a pterygotid would in turn signify that *all* eurypterine clades were established by the Early Ordovician. However, we emphasize that it is currently not possible to unequivocally make this identification based on cuticular ornament alone, and hence, currently, the presence of pterygotids in the Early Ordovician must remain as a plausible, yet unconfirmed possibility. In this context, the recent description of the possible adelophthalmid *Archopterus anjiensis* [102] from the Hirnantian (Late Ordovician) of China is significant, as adelophthalmids constitute the sister group of pterygotoids [5,14,15,21], implying that pterygotoids were present by at least the Late Ordovician.

Considering that Eurypterida either is the sister group to all Arachnida [14,15,21–23] or is contained within Merostomata, which together with Ricinulei forms the sister group to Arachnopulmonata [24,25], an early, likely Cambrian origin and major diversification for eurypterids by necessity implies an equally early origin either for arachnids as a whole or for ricinuleids, arachnopulmonates and several ‘apulmonate’ groups, respectively. This realization further expands an already significant gap in the fossil record of these groups [103], with classical Arachnida and Arachnopulmonata only making their first appearance in the Telychian (Llandovery, Silurian) with the early scorpion *Dolichophonus loudonensis* [104], while the earliest ‘apulmonate’ clades Acariformes [105] and Opiliones [106] appear in the Pragian (Early Devonian), and Ricinulei [107] first turn up in the early Bashkirian (Early Pennsylvanian, Carboniferous). The reasons for this extended hiatus are unclear, but might be explained by a delayed radiation, possibly combined with the persistent preferential occupation of environments with limited preservation potential (e.g. very shallow, near-shore settings). In this respect, it is interesting to note that most ‘apulmonates’ are relatively small to tiny, and it was previously suggested they may have independently invaded the land early on as soil arthropods [103].

It has been argued that Eurypterida likely originated in Laurentia, and that the subsequent evolutionary history of this group was mainly confined to Laurussia (the amalgamation of Laurentia, Baltica and Avalonia) during the Silurian and Devonian [7]. However, Lamsdell *et al.* [34] first asserted that eurypterids may have first evolved off Gondwana during the Ordovician and subsequently spread to Laurussia during the Silurian (see also [93]). The new discoveries from Fezouata, which include the first African record of carcinosomatids, provide additional support to this proposal. The increasing number of diverse Ordovician eurypterids (electronic supplementary material, table S5) discovered outside of Laurussia [34,102,108] and the fact that the oldest mixopterid genus, *Terropterus* [109], hails from the Llandovery of China, together with the realization that euchelicerates more generally may have had their first radiation off Gondwana, warrant a re-evaluation of the hypothesis for a Laurussia-centric evolutionary model for eurypterids. Historically, Laurussia is palaeontologically one of the most well characterized and collected areas on Earth [110], and it appears likely that the signal suggesting an evolutionary history centred in Laurussia is in large part due to collecting bias. Now that, finally, other areas of the globe are starting to be scrutinized to the same degree as Laurussia has been for the past two centuries, the true story of chelicerate origins and evolution may finally start to emerge from the mists of deep time.

## 4. Methods

Image stacks of specimens were photographed dry and wet under cross-polarized lighting [111,112] using various full-frame digital mirrorless interchangeable lens cameras. A detailed discussion of the photographic techniques and equipment employed and of the processing and post-processing of the images is provided in the electronic supplementary material.

## Data Availability

All studied specimens prefixed with NM S are held at the Natural History Museum of the National Museum in Prague (Přírodovědecké muzeum, Národní muzeum v Praze), Czech Republic. Material prefixed by SC is curated in the West Bohemian Museum in Pilsen (Západočeské muzeum v Plzni), Czech Republic. Material with the prefix MCZ.IP. resides in the Invertebrate Paleontology collections of the Museum of Comparative Zoology of Harvard University, in Cambridge, MA, USA. Detailed locality data, including GPS coordinates of the sites, are curated with the Fezouata specimens in their respective collections. Additional information on the geological setting and preservation of the Fezouata fossils is provided in the electronic supplementary material. For comparative purposes, Silurian carcinosomatid specimens in the collections of the American Museum of Natural History (AMNH) in New York City, NY, USA and the Geological Survey of Canada (GSC) in Ottawa, Ontario, Canada were inspected and photographed for documentation and inclusion in the electronic supplementary material, figures. Supplementary material is available online [113].
